# Bundling Colorectal Cancer Screening Outreach with Screening for Social Risk in Federally Qualified Health Centers: A Stepped-Wedge Implementation-Effectiveness Study

**DOI:** 10.1007/s11606-024-08654-5

**Published:** 2024-02-08

**Authors:** Gina R. Kruse, Sanja Percac-Lima, Marjanna Barber-Dubois, Madeline E. Davies, Daniel A. Gundersen, Oanh Ho, Lynette Mascioli, Mehezbin Munshi, Sarah Perry, Deepinder Singh, Annette Thomas, Karen M. Emmons, Jennifer S. Haas

**Affiliations:** 1 Division of General Internal Medicine, University of Colorado School of Medicine, Aurora, CO USA; 2 Division of General Internal Medicine, Massachusetts General Hospital, Boston, MA USA; 3 Harvard Medical School, Boston, MA USA; 4 Manet Community Health Center, Quincy, MA USA; 5 Kraft Center for Community Health, Massachusetts General Hospital, Boston, MA USA; 6 Survey and Qualitative Methods Core, Dana Farber Cancer Institute, Boston, MA USA; 7 Department of Social and Behavioral Sciences, Harvard T.H. Chan School of Public Health, Boston, MA USA; 8 Harbor Health Services, Inc., Mattapan, MA USA; 9 Massachusetts League of Community Health Centers, Boston, MA USA; 10 Larner College of Medicine, University of Vermont, Burlington, VT USA; 11 Brockton Neighborhood Health Center, Brockton, MA USA

**Keywords:** colorectal cancer, screening, community health centers, social determinants of health, implementation science.

## Abstract

**Background:**

Bundling is combining individual interventions to meet quality metrics. Bundling offers of cancer screening with screening for social determinants of health (SDOH) may enable health centers to assist patients with social risks and yield efficiencies.

**Objective:**

To measure effects of bundling fecal immunochemical testing (FIT) and SDOH screening in federally qualified health centers (FQHCs).

**Design:**

Clustered stepped-wedge trial.

**Participants:**

Four Massachusetts FQHCs randomized to implement bundled FIT-SDOH over 8-week “steps.”

**Intervention:**

Outreach to 50–75-year-olds overdue for CRC screening to offer FIT with SDOH screening. The implementation strategy used facilitation and training for data monitoring and reporting.

**Main Measures:**

Implementation process descriptions, data from facilitation meetings, and CRC and SDOH screening rates. Rates were compared between implementation and control FQHCs in each “step” by fitting generalized linear mixed-effects models with random intercepts for FQHCs, patients, and “step” by FQHC.

**Key Results:**

FQHCs tailored implementation processes to their infrastructure, workflows, and staffing and prioritized different groups for outreach. Two FQHCs used population health outreach, and two integrated FIT-SDOH within established programs, such as pre-visit planning. Of 34,588 patients overdue for CRC screening, 54% were female; 20% Black, 11% Latino, 10% Asian, and 47% white; 32% had Medicaid, 16% Medicare, 32% private insurance, and 11% uninsured. Odds of CRC screening completion in implementation “steps” compared to controls were higher overall and among groups prioritized for outreach (overall: adjusted odds ratio (aOR) 2.41, *p* = 0.005; prioritized: aOR 2.88, *p* = 0.002). Odds of SDOH screening did not differ across “steps.”

**Conclusions:**

As healthcare systems are required to conduct more screenings, it is notable that outreach for a long-standing cancer screening requirement increased screening, even when bundled with a newer screening requirement. This outreach was feasible in a real-world safety-net clinical population and may conserve resources, especially compared to more complex or intensive outreach strategies.

**Clinical Trials Registration:**

NCT04585919

**Supplementary Information:**

The online version contains supplementary material available at 10.1007/s11606-024-08654-5.

## INTRODUCTION

Gaps in implementation of evidence-based interventions pervade nearly all cancer prevention strategies, creating a situation where preventable inequities are tolerated.^1–3^ Although there are few racial/ethnic disparities in cancers with low survival rates, survival curves diverge as treatability increases and differences are greatest for cancers that can be detected early and treated successfully.^4–6^

In Massachusetts, despite broad coverage and healthcare infrastructure, cancer is the leading cause of death among residents and substantial inequities in incidence and outcomes exist in the state.^7,8^ Black non-Hispanic residents have the highest percentage of CRC diagnosed at distant stage (26% of CRC, vs. 20% among White residents), and in 2020, 13.7 CRC deaths/100,000 were measured among non-Hispanic Black residents (vs. 10.4 deaths/100,000 among White residents).^9,10^.

The need for equitable and efficient methods of delivering cancer screening services became more acute with the pandemic. One analysis predicted 10,000 excess deaths nationally from CRC and breast cancer would result from the pandemic.^11^ Federally qualified health centers (FQHCs) face persistent gaps in preventive care delivery and have been slower to recover from pandemic-attributable gaps compared to large integrated delivery systems.^12,13^ Addressing these gaps requires innovation and recognition of the social and cultural environments of FQHCs.

As new screening studies become available, such as low-dose CT, and attention to behavioral and social risks grows, the number of screening activities health systems are required to do has grown. Resource-constrained settings such as FQHCs are already struggling under the burden of achieving multiple screening metrics to meet the needs of their complex populations. Models for “bundled” screening, linking one cancer screening test with other services, have gained attention for their potential as an effective strategy for delivering services in settings where multiple, parallel workflows for individual screening interventions may not be possible due to capacity constraints.^14,15^ A recent article exploring studies of integrated interventions in cancer control defined blended and bundled interventions, noting that blended interventions integrate two evidence-based interventions into a single harmonized intervention while bundling combines multiple distinct behaviors simultaneously or sequentially.^16^ Examples include linking an offer of fecal immunochemical testing (FIT) to delivery of flu vaccines which doubled the odds of CRC screening.^17^ A pilot study among uninsured Latino women found that pairing CRC education and FIT screening with mammography was associated with almost 90% completion of FIT.^18^

Building on this work, we designed a bundle combining offers of CRC screening using FIT with screening for social determinants of health (SDOH). SDOH are “the conditions in the environments in which people live, learn, work, play, worship, and age.”^19^ SDOH, such as food insecurity or housing instability, are negatively associated with cancer screening behaviors.^20,21^ Plus, SDOH screening is an FQHC priority required by Medicaid every 12 months.^22–24^ FIT is commonly used in FQHC settings and is important for populations who face barriers to colonoscopy (e.g., need for an escort, time off work).^25^

We tested the bundled screening offer within the infrastructure of our Implementation Science Center for Cancer Control Equity (ISCCCE).^26,27^ ISCCCE is a partnership between the Harvard Chan School of Public Health, Massachusetts General Hospital Kraft Center for Community Health, Massachusetts League of Community Health Centers (Mass League, the primary care association for FQHCs), and Azara Healthcare LLC, makers of a population health program used by FQHCs and Mass League for quality reporting (Azara Data Reporting and Visualization System [DRVS]).^26,28^ We aimed to test the effects of bundled offers of FIT and SDOH screening in a real-world, resource-constrained care environment among adult FQHC patients at average risk for CRC on completion of CRC screening by any guideline recommended method and documented SDOH screening.

## METHODS

Using a cluster-randomized stepped-wedge trial, four Massachusetts FQHCs were randomized to start implementation of a bundled outreach intervention over 8-week “steps” between 12/2020 and 11/2021 (Table 1). Patients were eligible if they were aged 50 to 75 years and overdue for CRC screening, had a Uniform Data System (UDS)-qualified visit^29^ in the past 2 years, and were average risk for CRC and therefore appropriate for FIT screening. Eligibility was based on the 2016 U.S. Preventive Services Task Force (USPSTF) CRC screening guidelines in place at the study start, prior to the 2021 update expanding screening to ages 45–49 years.^30^ Average CRC risk was defined as age-eligible adults without family history or personal history of CRC, precancerous polyps, inflammatory bowel disease, or heritable cancer syndromes. The bundle combined the offer of CRC screening by FIT, a USPSTF Grade A recommendation, with an offer of SDOH screening, an annual Medicaid requirement.^21,24,30^ Standard care at FQHCs included provider-driven CRC screening plus population health monitoring of CRC screening. Standard care SDOH screening was conducted during rooming processes or by providers during visits.

**Table 1:**
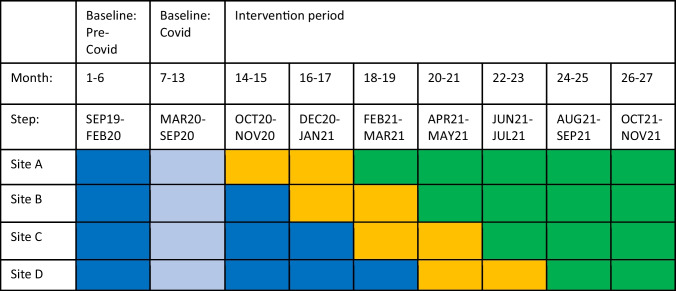
Stepped-Wedge Design

Dark blue, baseline/control step; light blue, excluded early COVID-19 pandemic step; yellow, intervention step; green, adaptation step

**Figure 1:**
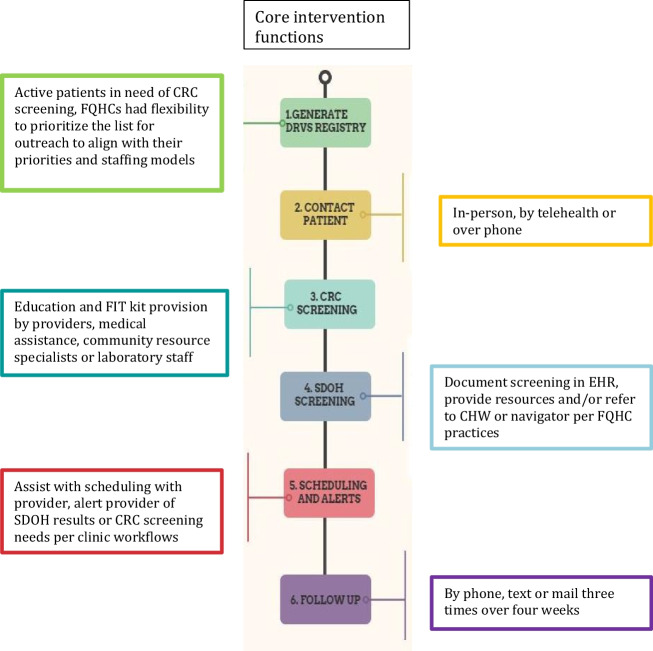
Intervention elements.

FQHC implementation teams were trained in core functions (Fig. 1) as well as flexible elements that could take on different forms to match FQHC workflows, resources, and team structures. For example, all FQHCs were required to generate a registry of eligible patients using population health data but had flexibility in selecting individuals to prioritize for outreach. With thousands of eligible patients, FQHCs determined who to outreach first based on priorities and staffing models. Teams were trained to offer both screenings but patient participation in both was not a requirement for delivery. This aligns with previously tested bundled interventions such as the CDC’s CRC Program which offered multiple cancer screenings and found that not all patients were open to discussing screenings other than CRC.^31^

### Implementation Processes

The implementation strategy consisted of external facilitation with the ISCCCE implementation team, internal facilitation by a team convened by each FQHC, plus training from Mass League in the Azara DRVS system.^26^ Each FQHC implemented the study intervention over two phases: (1) initial implementation (months 1–4); and (2) intervention adaptation guided by data to address inequities in reach and effectiveness (months 5–8).

The implementation processes are described elsewhere.^32^ Briefly, FQHCs were provided with external, guided facilitation from the ISCCCE Implementation Lab team.^33^ Facilitation meetings comprising internal facilitation teams and the external Implementation Lab were recorded with permission and involved weekly or bi-weekly meetings for the first 4 months, and monthly thereafter for the 8-month implementation. Mass League provided training with DRVS to support creation of registries and summary data to guide adaptation (Table 1).

In this real-world setting, incorporating flexibility ensured feasibility for each site and leveraged their unique resources. The bundled strategy was delivered by FQHC staff, not research staff, and used FQHC resources. Descriptions of the unique elements of each FQHC’s implementation strategy were derived from facilitation meeting notes.

### Outcome Measures

The primary outcome measures were FIT and SDOH screening rates overall at the FQHCs including both screening done following FIT-SDOH outreach and all other screening happening in the FQHC among eligible patients. Outcomes were measured using Azara DRVS which maps onto electronic health record (EHR) fields. CRC screening was defined according to UDS as screening completion by any guideline-based method (e.g., completed colonoscopy, sigmoidoscopy, or lab result for stool-based screening).^34^ All four FQHCs had implemented SDOH screening in rooming workflows but used different SDOH screening tools based on their local accountable care agreements and population needs.^22^ The tools included PRAPARE, a modified version of PRAPARE tailored for local resources, and novel tools embedded in the EHR.^35^ All SDOH tools screened for housing, food, transportation, and utilities and some also screened for other elements such as employment, education, or interpersonal violence. We examined three elements used by all FQHCs: housing instability, food insecurity, and transportation needs. SDOH screening was defined a priori as documentation of screening for one or more of these three elements.

Secondary outcomes measured implementation processes using summary data on outreach efforts by FQHCs. These included the number of FIT kits distributed by the outreach team and number of reminders per kit distributed and per kit returned.

### Statistical Analysis

We compared screening rates in intervention and control steps by fitting generalized linear mixed effects models. We included random intercepts for FQHCs and patients to account for clustering of observations within FQHCs and multiple measurements among patients as well as random intercepts for FQHC by step to allow for different random effects across time within the same cluster. To isolate the intervention effect, models were adjusted for secular trend by including step as a categorical variable, age during the step, race/ethnicity, language, household income (% Federal Poverty Level), insurance, comorbidities, and cumulative number of healthcare visits. Baseline control observations used data in the four FQHCs over the 6 months prior to COVID-19 pandemic shutdowns (March 2020). We used multiple imputation to address missing data in race/ethnicity (11.2% missing), language (6.2% missing), and insurance (7.5% missing). We used 30 imputed datasets and combined estimates using Rubin’s rule.^36^ *p* values were calculated based on a two-sided *t*-test with degrees of freedom defined as cluster × periods minus the number of cluster-level parameters, adjusted due to multiple imputation. We compared screening rates overall and among groups prioritized by each FQHC. Not all prioritizations selected by sites were measurable in EHR data; in sites with prioritizations that were not in the EHR, we used their overall population for analysis. We further examined whether odds of screening for the first and second phases representing initial implementation and adaptation were different. All analyses were conducted in R using the glmmTMB package for fitting generalized linear mixed-effects models and the mice package for multiple imputation.^37–39^

## RESULTS

### Implementation Processes

Facilitation notes demonstrated that FQHC sites operationalized their implementation processes to match their unique infrastructure, workflows, capacity, and pandemic-related staffing demands (Table 2). Two FQHCs used population health approaches that involved outreaching patients by phone between visits. Of these, one FQHC prioritized groups with screening rates below the clinic average and the second prioritized patients who had screened with FIT in the past. Outreach efforts included patients who spoke languages the internal facilitation teams could support. Two FQHCs integrated outreach within established teams and visit-based workflows; one added bundled screening to the activities of community health workers (CHWs) working on non-communicable disease management and the second embedded bundled FIT-SDOH in pre-visit planning workflows and prioritized patients overdue for screening with a visit in the next 3 months. The impact of the pandemic on implementation was apparent in several ways. All sites experienced project staff turnover and most also experienced staff redeployment for COVID-19 activities. This turnover slowed or paused FIT-SDOH outreach while staff were redeployed, or new staff were onboarded. Other impacts included visit volume fluctuations with some FQHCs temporarily pausing primary care. Additionally, there were times when colonoscopy was not available to FQHC patients for screening or evaluation of positive FIT results.

**Table 2:** Implementation Approach by Site

FQHC	No. of staff on implementation team	Bundled FIT/SDOH outreach approach		
		On-site visit	Virtual visit	Outreach not linked to visit
A	6	Y		Y
B	4			Y
C	7	Y	Y	Y
D	5	Y		

### Screening Outcomes

Across the four sites, a total of 34,588 individual patients met eligibility criteria for CRC screening during the study period. Table 3 shows characteristics of eligible individuals. The median age was 61 years (IQR 55, 67) with 54% female, 47% white, non-Latino, 20% Black, non-Latino, 11% Hispanic or Latino, 10% Asian, and 0.9% another race/ethnicity. English (64%) was the most common primary language, followed by Portuguese (6.9%), Spanish (6.1%), and Cape Verdean (5.3%). Prevalence of comorbidities is shown in Supplemental Figure A.

**Table 3:** Participant Characteristics (*N* = 34,588)

	*n* (%)
Age in years (median, IQR)^1^	61 (55, 67)
Sex assigned at birth	
Female	18,550 (54%)
Male	16,024 (46%)
Other	14 (< 0.1%)
Race/ethnicity	
White, non-Latino	16,298 (47%)
Black, non-Latino	6752 (20%)
Hispanic/Latino	3818 (11%)
Asian	3510 (10%)
Other, non-Latino	295 (0.9%)
AI/AN/PI	153 (0.4%)
Not in EMR/unknown	3762 (11%)
Primary language	
English	22,065 (64%)
Portuguese	2393 (6.9%)
Spanish	2115 (6.1%)
Cape Verdean	1817 (5.3%)
Vietnamese	1110 (3.2%)
Khmer	1089 (3.1%)
Haitian Creole	1073 (3.1%)
Arabic	178 (0.5%)
Other	997 (2.9%)
Not in EMR/unknown	1751 (5.1%)
Insurance source	
Private insurance	11,159 (32%)
Medicaid	9734 (28%)
Medicare	5601 (16%)
Uninsured	3668 (11%)
Other public	789 (2.3%)
Dual-eligible Medicare and Medicaid	325 (0.9%)
Not in EMR/unknown^2^	3312 (9.6%)
Number of visits by end of project period^3^	2 (0, 6)

*AI/AN/PI* American Indian/Alaska Native/Pacific Islander, *EMR* electronic medical record

^1^Median (IQR)

^2^Insurance field non-missing but not mappable into structured categories

^3^Cumulative number of visits by end of project period, modeled as cumulative number of visits from start of project to end of step

A total of 3194 unique patients completed CRC screening and 16,613 completed SDOH screening during the study. Table 4 shows odds of screening in implementation steps compared to control steps, controlling for secular trend, patient demographics, and comorbidities. Overall, the odds of CRC screening were more than twice as likely in implementation steps compared to control steps (OR = 2.41, *t*_(df = 19.25)_ = 3.16, *p* = 0.005) after controlling for secular trend and patient characteristics and comorbidities. This corresponds to an average marginal effect of 1.4-percentage point increase (95% confidence interval, 0.3–2.3%) across the study period. Among groups prioritized in the first implementation phase for outreach, there was also a higher odds of CRC screening in implementation steps compared to control steps (OR = 2.88, *t*_(df=19.25)_ = 3.65, *p* = 0.002). CRC screening rates were not different in the second phase adaptation steps compared to first implementation phase steps (OR = 0.58, *t*_(df=19.25)_ = 1.47, *p* = 0.16). The odds of SDOH screening were not significantly different in implementation steps compared to control steps overall (OR = 0.64, *t*_(df = 19.25)_ = 1.36, *p* = 0.189) or within prioritized groups (OR = 0.51, *t*_(df=19.25)_ = 1.78, *p* = 0.09).

**Table 4:** Odds of CRC and SDOH Screening Completion Overall

	Odds ratio^a^	*t*-value (degrees of freedom)^b^	*p* value
CRC screening completion			
Overall^c^	2.41	3.16 (19.25)	0.005
Priority population^d^	2.88	3.65 (19.25)	0.002
SDOH screening			
Overall^c^	0.64	1.36 (19.25)	0.189
Priority population^d^	0.51	1.78 (19.25)	0.09

^a^Adjusted for age, race/ethnicity, primary language, insurance source, comorbid conditions, cumulative number of FQHC visits during project period

^b^Two-sided* t*-test with degrees of freedom = cluster × periods – no. of cluster level parameters, adjusted for multiple imputation

^c^Overall: All patients meeting eligibility

^d^Priority population: Subset of patients in priority outreach groups for each FQHC

Secondary implementation process outcomes reported by FQHCs during facilitation showed 5103 patients were outreached for FIT-SDOH and 4404 FIT were distributed. The mean number of reminder calls per kit distributed during outreach was 0.87 (standard deviation (SD) 0.43) and the mean number of calls per FIT return was 11.7 calls (SD 7.43).

## DISCUSSION

This study adds to the growing literature on bundled screening strategies. We bundled FIT and SDOH because our FQHC partners wanted to increase screening rates for both and bundling was hypothesized as a way to do this within local staffing constraints. We found the effect of the bundled FIT-SDOH significantly increased CRC screening compared to usual practices with a relative effect similar to the effect found in meta-analyses of CRC outreach programs.^40^ In the challenging circumstances of the pandemic, particularly for FQHCs with substantial overall decreases in cancer screening during 2020–2021,^13^ even modest success screening the unscreened is meaningful. Further, a contribution of this work is the study of an approach for addressing different kinds of patients’ needs in an efficient way—that is, providing support for cancer screening as well as social needs screening that can perhaps address more immediate patient concerns. With the significant staffing constraints that FQHCs face, being able to address two screening topics and maintain and even improve CRC screening uptake is highly relevant and important. This simple outreach strategy to improve a long-standing screening requirement, CRC screening, remained effective at increasing cancer screening even when bundled with a newer screening requirement among complex patient populations. This bundling strategy which improved cancer screening without negatively affecting SDOH screening may produce resource savings especially relevant in safety-net care systems.

We showed significant increases in CRC screening overall in the FQHCs and among patients prioritized for outreach. While some may deem it obvious that more outreach increases screening, not all outreach succeeds. The intensity or complexity of outreach may impact the real-world feasibility or effectiveness in care settings serving low-income populations.^41^ There are often assumptions that complex patient populations require more intensive interventions to produce change. We demonstrated that a simple outreach bundle was both feasible and produced cancer screening improvements in real-world, complex FQHC populations. This increase in cancer screening is especially encouraging from an equity perspective given that one of our FQHCs prioritized marginalized groups with structural barriers to care and screening rates that were lower than the average screening rate overall in the health center. In facilitation meetings, we learned that many patients in the lower-than-average screening groups faced barriers like loss of insurance that required additional time from the outreach team before engaging patients in CRC screening. The observed number of reminders per FIT distributed and per FIT returned using bundled outreach underscores the human resources needed for this model. The large number of calls per FIT kit return has practical implications. It also creates opportunities to support patients with other needs. Our FQHC partners noted that calls often led to healthcare questions that could be addressed between visits. Automated approaches to CRC screening outreach, like text message reminders, have demonstrated promising improvements in CRC screening^42,43^ but may be less able to connect patients with assistance for some barriers like insurance changes. Future work could combine automated and live interactions for CRC screening with a modified pathway for patients requiring human assistance.

Bundled FIT-SDOH did not increase both screenings. This bundled intervention differs from sequential cancer screening pairings such as FLU-FIT or offering lung screening at mammography, where a patient is engaged in prevention (e.g., influenza vaccination or mammogram) when a second service is offered.^17,18,44^ The FIT-SDOH bundle relied on simultaneously extending offers for two services. Further research examining bundle variations, such as offering FIT for patients engaging with social resources like food pantries, may yield benefits.

Reasons for the lack of increase in SDOH screening may include patients declining to be screened, or staff not asking. The EHR context may have contributed. One FQHC underwent an EHR transition late in their implementation period and noted the detail and priority of SDOH screening were improved with their new system. Staff and patient time constraints are another likely influence. If the staff usually tasked with SDOH screening were additionally tasked with CRC screening outreach, they may have less bandwidth for usual care SDOH screening. FQHCs also noted that time-constrained patients often wanted to get to the health-related part of phone calls and not spend time on SDOH. The pandemic was also a factor. For some, the rollout of telehealth workflows did not include medical assistant rooming activities, the point when SDOH screening occurred in usual care. Plus, there was the great resignation and pandemic staffing constraints.^45^ Our study sample did not exclude those recently screened for SDOH. The number of patients who had documented SDOH screening during the study exceeded the number outreached for FIT-SDOH, demonstrating the extent of SDOH screening happening as standard care in FQHCs. Staff may have focused on CRC screening if SDOH was recently addressed. Patients may have declined SDOH screening if they received assistance elsewhere, such as at COVID-19 vaccination sites. It is possible that the offer to discuss social risks helped to build rapport by acknowledging challenging social circumstances. FQHC partners endorsed this notion, noting that the offer of SDOH screening often prompted further conversations about well-being, even among patients who declined SDOH screening. Our study also adds to emerging evidence examining the integration of SDOH support with cancer screening. A systematic review of the impact of SDOH interventions on cancer screening showed that among cancer screening types, SDOH interventions had the smallest effects on CRC screening.^46^ Another review found that SDOH interventions that aimed to improve cancer screening were cost-effective.^47^ Our team demonstrated the feasibility, acceptability, and appropriateness of our facilitation processes and showed improved CRC screening outcomes with bundled FIT-SDOH, but further research on sustainability or scalability is needed.^32^

### Limitations

Our clustered stepped-wedge design is vulnerable to major contextual events like the pandemic. Unlike integrated healthcare delivery systems, most FQHCs rely on external partners for endoscopy and laboratory services. This means that outcome data derived from the EHR depended on screening data being collected from outside providers and entered into the local EHR. We did not compare the combined FIT-SDOH strategy with individual outreach for FIT or SDOH alone. However, bundling may have more real-world relevance than siloed screening strategies in settings with capacity constraints limiting their ability to add additional individual service workflows.^16^ The study was not blinded and desirability bias may have influenced facilitation interactions. The number of calls achieved by participating FQHCs may not be feasible for some practices. Measurement of sustainability was beyond the scope of this study. Despite these limitations, the increase in cancer screening achieved by participating FQHCs represents a finding worthy of further study.

## CONCLUSIONS

In the context of implementing a bundled intervention, cancer screening increased. Screening for SDOH did not decrease when FIT-SDOH was added to busy workflows. Future studies are needed to examine the relationship between SDOH and cancer screening and the impact of bundled strategies on staff time and efficiency to improve our understanding of the costs and benefits of bundled services. This outreach strategy increased cancer screening among complex FQHC populations. Bundling cancer screening and SDOH screening warrants consideration as an outreach approach in resource-constrained settings.

### Supplementary Information

Below is the link to the electronic supplementary material.

Supplementary file1 (DOCX 88 KB)

## Data Availability

Data generated through Center activities will be shared in accordance with Cancer Moonshot funding policies.
