# Supplementary figures and images for: Bundling Colorectal Cancer Screening Outreach with Screening for Social Risk in Federally Qualified Health Centers: A Stepped-Wedge Implementation-Effectiveness Study
Source: J Gen Intern Med. 2024 Feb 8;39(7):1188–95. doi: 10.1007/s11606-024-08654-5 (PMC11116362; doi:10.1007/s11606-024-08654-5)

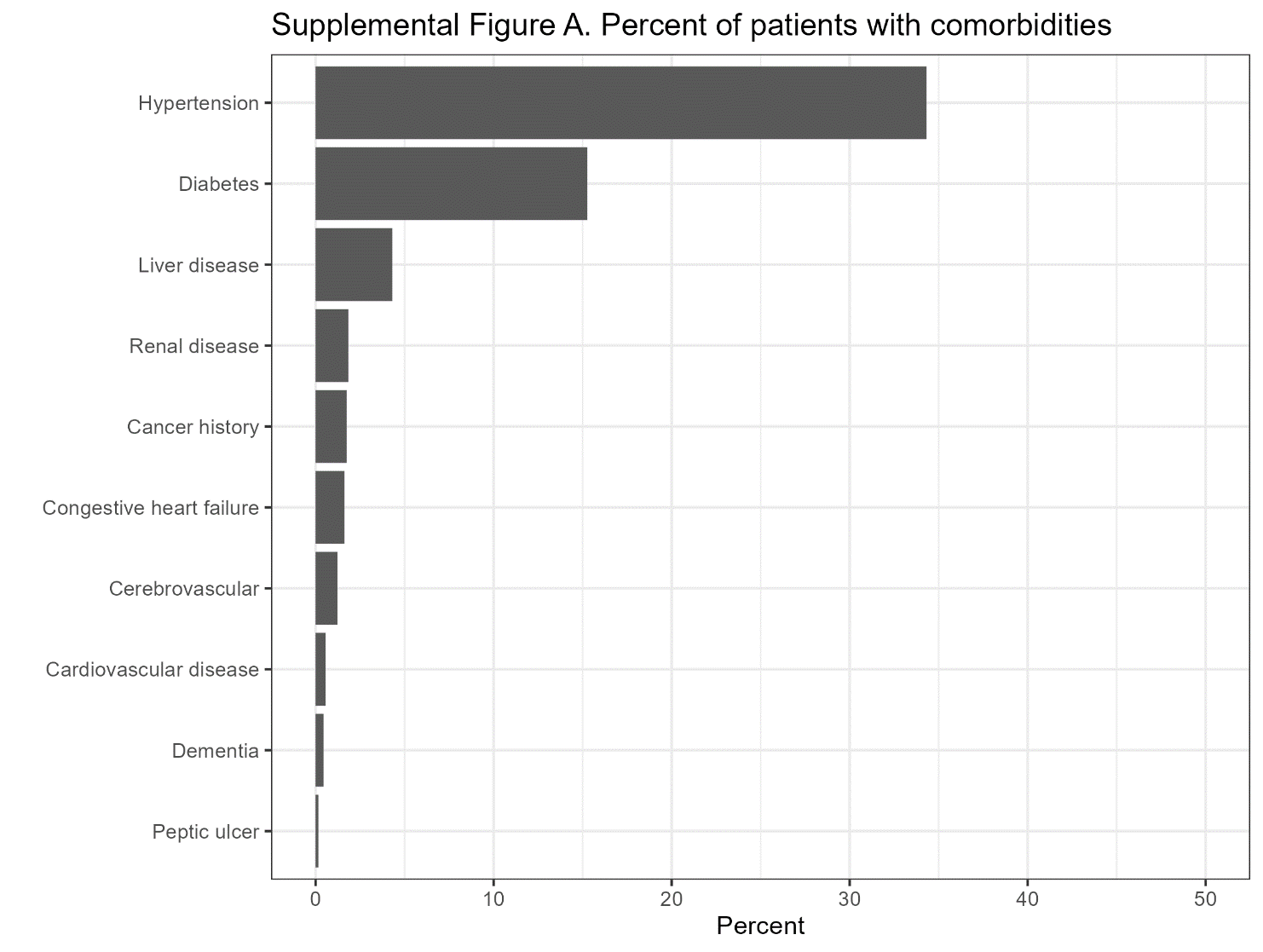

Supplement: Supplementary file 1 — Supplementary file1 (DOCX 88 KB) [file 11606_2024_8654_MOESM1_ESM.docx]
